# Blood Lead Levels in Children Aged 0–6 Years Old in Hunan Province, China from 2009–2013

**DOI:** 10.1371/journal.pone.0122710

**Published:** 2015-04-01

**Authors:** Jun Qiu, Kewei Wang, Xiaoli Wu, Zhenghui Xiao, Xiulan Lu, Yimin Zhu, Chao Zuo, Yongjia Yang, Youjie Wang

**Affiliations:** 1 Department of Maternal and Child Health, School of Public Health, Tongji Medical College, Huazhong University of Science & Technology, Wuhan 430030, China; 2 Department of Children’s Intensive Research Center, Hunan Children’s Hospital, Changsha 410007, China; 3 Department of Obstetrics and Gynecology, Maternal And Child Health Hospital Of Hunan Province, Changsha 41000, China; Institute for Health & the Environment, UNITED STATES

## Abstract

**Objectives:**

The aim of this study is to describe blood lead levels (BLLs) and the prevalence of elevated blood lead levels (EBLLs) in children aged 0–6 years old and to analyze the BLL trend in children from 2009 to 2013 in China.

**Methods:**

A total of 124,376 children aged 0–6 years old were recruited for this study from January 1^st^ 2009 to December 31^st^ 2013. Their blood lead levels were analyzed using atomic absorption spectrometry.

**Results:**

The median BLL was 64.3 μg/L (IQR: 49.6–81.0), and the range was 4.3–799.0 μg/L. Blood lead levels were significantly higher in boys (66.0 μg/L) than in girls (61.9 μg/L) (*P*<0.001). The overall prevalence of BLLs≥100 μg/L was 10.54% in children aged 0–6 years in Hunan Province. Between 2009 and 2013, the prevalence of EBLLs (≥100 μg/L) decreased from 18.31% to 4.26% in children aged 0–6 years and increased with age. The prevalence of EBLLs has dramatically decreased in two stages (2009–2010 and 2012–2013), with a slight fluctuation in 2010 and 2011.

**Conclusions:**

Both BLLs and the prevalence of EBLLs in children aged 0–6 years old declined substantially from 2009 to 2013 in Hunan Province; however, both remain at unacceptably high levels compared to developed countries. Comprehensive strategies are required to further reduce blood lead levels in children.

## Introduction

It is well known that there is no safe blood lead level (BLL) in children. Many studies have demonstrated that lead exposure is related to the risk of behavioral and intellectual deficits, hypertension, nephropathy and infertility [1–7]. The National Toxicology Program (NTP) concluded that the presence of lead in blood at BLL<100 μg/L was associated with delayed puberty and poor cognitive performance and with a lower intelligence quotient (IQ) in the United States [8]. Liu JH et al. showed that Chinese children with BLLs ≥80 μg/L have lower scores than those with BLLs<80 μg/L in IQ tests and academic performance [9]. Children, especially younger children, are at greatest risk for elevated BLLs. Some studies have demonstrated that children appear to have approximately four to five times higher lead absorption than adults [10], and the detoxification capability of lead in adults was three times higher than in children [11].

Burning fossil fuels with lead, wall paints with lead, drinking water from lead containing pipes and industrial manufacturing processes are the major sources of lead pollution in air, water and soil. Lead in the air, water and dust can be absorbed by children through the respiratory tract and digestive tract. In Europe and the United States, control measures to regulate lead in petrol, paint, food and drinking water were implemented to reduce the lead exposure in daily surroundings since the 1970s. Blood lead levels in children have decreased considerably over the last several decades in the United States. In China, lead free gasoline was forcibly adopted by the Chinese Government to reduce lead exposure on July 1^st^ 2000. Since then, the prevalence of EBLLs in children dramatically decreased from 34% in 1995–2003 to approximately 24% in 2001–2007 [12]. With the unprecedented growth of China’s lead-acid battery industry from the electric bike, automotive, and photovoltaic industries, lead exposure remains an important public health concern [13]. The aim of our study was to analyze the blood lead level (BLL) in children aged 0–6 years old from 2009–2013 and to reveal the trend of BLLs in Chinese children.

## Materials and Methods

### Ethics statement

The study was approved by the Ethics Committee of Hunan Children’s Hospital. Informed written consent was also obtained from a legal guardian of each child involved in our study before data collection. All of the samples were sent to the clinic laboratory to detect the BLLs, and all data collected from the participants were fully anonymous.

### Study subjects

The Children who received regular health checkups at outpatient clinic of Hunan Children’s Hospital between Jan 1st 2009 and Dec 31st 2013 were recruited for the present study. All the children were residents of Hunan Province. Hunan Children’s Hospital is the largest tertiary hospital, providing service to a population of 71 million and a land area of 211,800 km^2^ in China. Hunan Province is located in central China and had a population of 222 million children in 2013.

Children who presented to the clinic for regular checkups and resided in Hunan Province were included in the study. 18 children with a typical symptom of lead toxicity such as seizures, neurological disorders and other symptoms were excluded in this study because these children who exposed to a severe lead poisoning outbreak in one area of Hunan Province in 2010. For any given year, a child was counted only once based children’s name, home address and the phone number of children’s parent. For a child with a confirmed EBLL among many tests in any given year, the highest value was considered as the blood lead level for the child. A total of 124,376 children were included in the study from 2009–2013. The annual subjects were 20,910, 22,802, 28,228, 28,278, and 24,158 children, respectively from the 2009 to 2013.

### Blood lead level determinations

Blood samples were taken from each child by trained staff into lead-free vacutainer tubes containing sodium heparin and were stored at 4°C prior to lead analysis. The blood lead level was analyzed using atomic absorption spectrometry (Z-2700 Hitachi Limited). The spectrometer, reagents and standard method were all provided by Hitachi Limited in Japan. Analysis of each sample was performed in duplicate, and the mean of both values was used as the final value. All reagents, glassware and sample collection devices were checked for lead contamination. The sample measurement was carried out in accordance with the manufacturer’s instructions. The technicians in our laboratory had received the training of a CDC (Center for Disease Control and Prevention of China)-administered quality-control program (Blood Lead Proficiency Testing Program) for the measurement of lead in blood sample. The lead measurement was carried out according to standard procedure provided by CDC of China. We defined BLLs≥100 μg/l as elevated BLLs (EBLLs) based on the guidelines by the Medical Administration Department of the Ministry of Health of China in 2006.

### Statistical analysis

Because BLLs were not normally distributed, the median and interquartile range were used to describe the blood lead level in children. The Mann-Whitney U test was used to compare the BLLs by gender. The Kruskal-Wallis test was used to compare the BLLs of different age groups. The percentage of EBLLs was calculated by age and gender. Differences between groups were assessed using the *χ* ^*2*^ test to evaluate the prevalence of EBLLs. The Spearman's rank correlation coefficient test was used to examine the temporal trends for BLLs, and the Cochran–Armitage test to examine the temporal trends for the prevalence of EBLLs. All analyses were performed using the Statistic Package for Social Sciences (SPSS) for Windows version 18.0. The figures were plotted using Microsoft Excel 2007. All of the statistical tests were two-sided. *P* values less than 0.05 were considered statistically significant.

## Results

### The median of the blood lead levels

**Fig 1:**
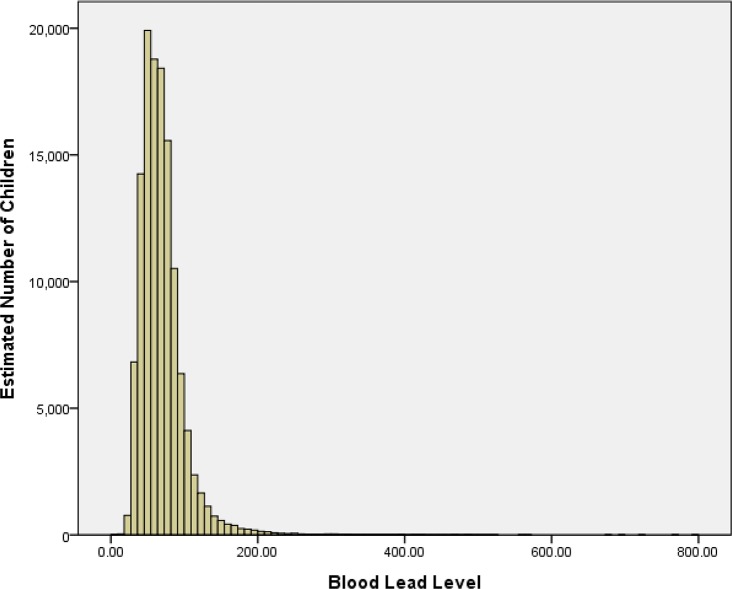
Blood lead levels in children aged 0~6 years living in Hunan Province in 2009–2013.

Of the 124,376 children enrolled in this study, 63.14% were boys. The average age was 3.2±2.4 years old. Fig 1 shows that the BLL has a positively skewed distribution. There were 13,112 (10.54%) children with elevated blood lead levels (≥100 μg/L) in this study, 12,247 (9.85%) children with values between 100 μg/L and 199 μg/L, 835 (0.67%) children with values between 200 μg/L and 449 μg/L, 27 (0.02%) children with values between 450 μg/L and 699 μg/L, and 3 children with values of more than 700μg/L.

For all children, the median BLL was 64.3 μg/L (IQR: 49.6–81.0), and the range was 4.3–799.0 μg/L. The median values were as follows: 73.0 μg/L (IQR: 53.0–92.0) in 2009, 63.0 μg/L (IQR: 47.5–82.0) in 2010, 66.0 μg/L (IQR: 51.4–81.8) in 2011, 65.0 μg/L (IQR: 51.0–79.0) in 2012 and 58.6 μg/L (IQR: 46.9–71.1) in 2013. Between 2009 and 2013, the median BLL decreased from 73.0 μg/L to 58.6 μg/L (r_s_ = -0.137, *P*<0.001). However, the median BLL slightly increased in 2011 and 2012 compared to 2010. The median BLL was 66.0 μg/L (IQR: 51.0–82.5) in boys and 61.9 μg/L (IQR: 48.0–78.0) in girls. The median BLL for boys was higher than girls, and the difference was statistically significant (*P*<0.001). Table 1 shows that the median BLL for boys was significantly higher than for girls in each year (*P*<0.001).

**Table 1:** Comparison of the BLLs in children for different genders from 2009 to 2013.

Years	Boys			Girls			*P*
	n	P_50_(P_25_~P_75_)	max	n	P_50_(P_25_~P_75_)	max	
2009	13243	75(54~94)	567	7667	70(51~89)	352	<0.001
2010	14431	65(49~84)	490	8371	60(46~79)	463	<0.001
2011	17714	68(53~84)	516	10514	63(49~79)	498	<0.001
2012	17805	67(52~80)	573	10473	63(49~76)	799	<0.001
2013	15333	60(48~72)	764	8825	57(46~69)	483	<0.001
Total	78526	66(51~83)	764	45850	62(48~78)	799	<0.001

Table 2 shows that the median BLL was lowest (50 μg/L) for children between 0 and 12 months old and highest (69.7 μg/L) for children aged 6 years. The median BLL increased with age in all subjects. The median BLL for boys was higher than for girls in each age group from 2009 to 2013. The median BLL increased with age in children from 2009–2010; however, from 2011 to 2013, the median BLL increased with age until the age of five and then decreased for six year olds. In boys, the median BLL increased with age from 2009 to 2010, while the median BLL increased with age until the age of five and then decreased for six year olds from 2011 to 2013. In girls, the median BLL increased with age until the age of five and then decreased for six year olds in two stages (2009–2010 and 2012–2013).

**Table 2:** Blood lead level (ug/l), by sex and age, in children aged 0 to 6 years living in China in 2009–2013.

Variants	0~		1~		2~		3~		4~		5~		6~	
	n	P_50_(P_25_~P_75_)	n	P_50_(P_25_~P_75_)	n	P_50_(P_25_~P_75_)	n	P_50_(P_25_~P_75_)	n	P_50_(P_25_~P_75_)	n	P_50_(P_25_~P_75_)	n	P_50_(P_25_~P_75_)
2009	2094	56(44~76)	4585	70(50~86)	3109	72(52~89)	2626	76(55~93)	2812	79(58~98)	2679	79(57~99)	3005	82(59~103)
Boys	1210	58(45~77)	2749	71(51~87)	1973	73(53~91)	1672	78(57~95)	1829	81(60~99)	1795	81(59~102)	2015	83(61~105)
Girls	884	55(43~75)	1836	67(49~85)	1136	70(50~87)	954	74(52~90)	983	75(55~94)	884	75(54~95)	990	78(56~98)
2010	2226	49(39~64)	4896	58(44~77)	3473	63(48~80)	2965	67(50~85)	2998	67(51~85)	3065	69(51~87)	3179	69(52~89)
Boys	1313	49(40~66)	2884	59(45~79)	2224	64(48~82)	1929	69(51~87)	1944	69(52~69)	2012	71(53~89)	2125	72(54~90)
Girls	913	48(39~63)	2012	56(43~74)	1249	60(46~78)	1036	62(48~82)	1054	65(49~82)	1053	67(49~85)	1054	64(49~82)
2011	3807	50(42~64)	5874	62(49~77)	4112	67(53~81)	3651	70(56~85)	3692	71(57~86)	3450	72(58~87)	3642	72(58~86)
Boys	2211	51(42~65)	3497	63(50~79)	2565	68(54~83)	2359	71(57~86)	2409	72(58~86)	2254	74(59~89)	2419	74(60~88)
Girls	1596	50.0(42.0~63)	2377	60(48~74)	1547	65(51~79)	1292	67(54~82)	1283	70(56~85)	1196	69(56~83)	1223	68(55~81)
2012	2512	51(41~66)	6919	60(47~74)	4120	66(52~79)	3729	68(54~81)	3758	69(56~82)	3589	70(57~83)	3651	69(57~81)
Boys	1465	51(42~67)	4060	60(48~75)	2581	68(54~80)	2378	69(55~82)	2469	70(57~83)	2396	72(59~86)	2456	71(59~84)
Girls	1047	50(41~65)	2859	59(46~73)	1539	64(50~77)	1351	66(53~78)	1289	67(54~79)	1193	68(55~79)	1195	66(54~77)
2013	1913	48(41.~59)	5268	54(45~66)	3576	59(46~71)	3350	61(48~73)	3447	62(49~73)	3283	63(50~74)	3321	62(50~74)
Boys	1129	48.4(42~59)	3143	55(45~67)	2237	59(47~72)	2134	63(49~74)	2269	63(50~74)	2232	64(51~75)	2189	63(51~76)
Girls	784	48.0(41~59)	2125	54(44~66)	1339	57(45~71)	1216	59(47~71)	1178	60(46~71)	1051	60(49~71)	1132	59(47~72)

### Elevated blood lead levels

**Fig 2:**
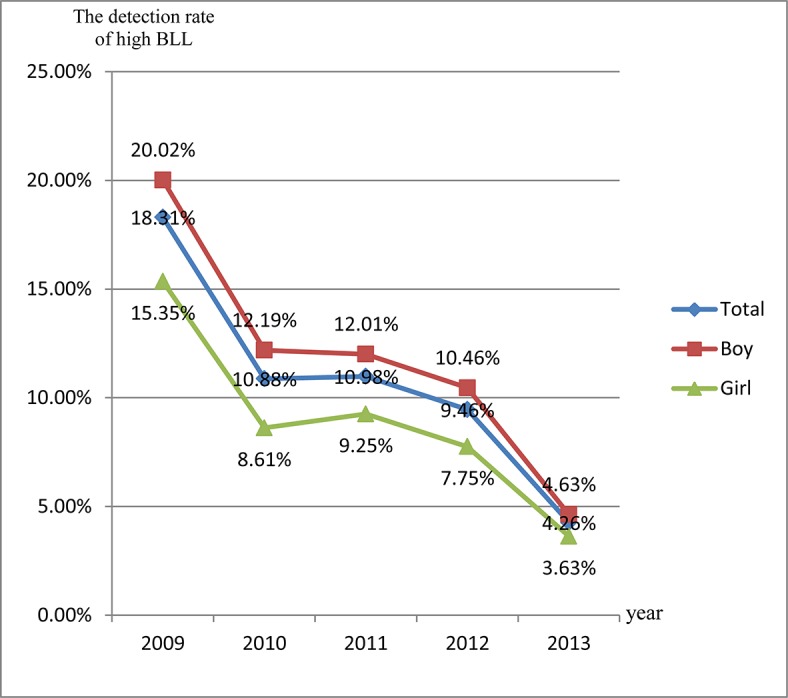
The detection rate of high BLL in different age groups from 2009 to 2013.

The overall prevalence of BLLs≥100μg/L was 10.54% in children aged 0–6 years. The prevalence of EBLLs was higher for boys (11.60%) than for girls (8.73%) from 2009 to 2013, and the difference was statistically significant (*χ* ^*2*^ = 252.71, *P*<0.001). Between 2009 and 2013, the prevalence of EBLLs decreased from 18.31% to 4.26% in children aged 0–6 years (*Z* = 43.41, *P*<0.001). Fig 2 shows that the prevalence of EBLLs dramatically decreased in 2009–2010 and 2012–2013. The prevalence of EBLLs decreased from 2009 to 2013 for boys and girls in this study. In each year, the prevalence of EBLLs was higher for boys than for girls between 2009 and 2013.

**Fig 3:**
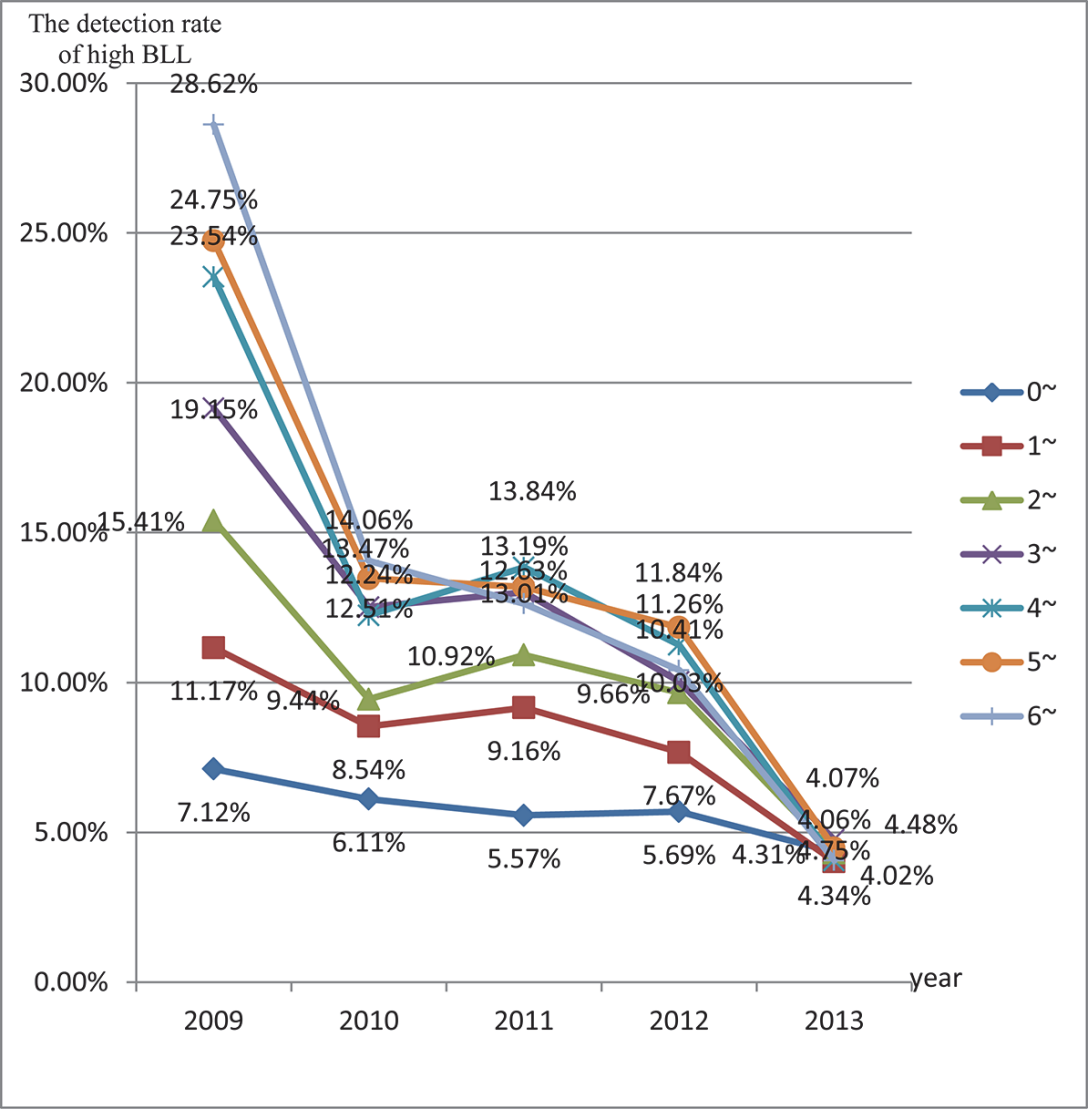
The detection rate of high BLL in different age groups from 2009 to 2013.

The prevalence of EBLLs was the lowest in children aged 0–12 months (5.76%), and the prevalence of EBLLs increased with age. In each year from 2009 to 2010, the prevalence of EBLLs increased with age. From 2011 to 2013, the prevalence of EBLLs increased with age until the age of five years and then decreased for six year olds (Fig 3). Between 2009 and 2013, the prevalence of EBLLs decreased among all age groups. Fig 3 shows that the prevalence of EBLLs was significantly reduced in 4–6 years old.

## Discussion

We analyzed BLL data for 124,376 children who presented to the clinic for regular checkups in Hunan Children’s Hospital from 2009 to 2013 and found that the median BLL for all children was 64.3μg/L. Both the BLLs and prevalence of EBLLs for boys were higher than for girls between 2009 and 2013. Overall, the BLLs and prevalence of EBLLs showed a clear downward trend from 2009 to 2013, but the trend showed a slight fluctuation from 2010 to 2012. The BLLs and prevalence of EBLLs increased with age in children in 2009 and 2010. The BLLs and prevalence of EBLLs increased with age until the age of five and then decreased for six-year-old children from 2011 to 2013.

In this study, the median BLL (64.3μg/L) and the prevalence of EBLLs (10.54%) for all children were different from other published literature in China. He K et al. analyzed articles published from 2004–2007 to assess the BLLs of Chinese children aged 0–14 years and found that the weighted mean BLL of 94,778 children was 80.7 μg/L and that 23.9% had an EBLL [12]. Li JM et al. showed that the mean BLL was 74.77 μg/L and that the prevalence of EBLLs was 20.05% in Changsha city among 1,431 children aged 0–6 years old [14]. The BLLs and prevalence of EBLLs in these previous studies were higher than in this study. There may be several explanations for this observation. First, besides leaded gasoline, other important sources of lead exposure, such as industrial emissions and lead-contaminated paints, have been gradually restricted by the government in recent years. In addition, the risk factors for high BLLs, such as eating (preserved eggs and canned food) and living habits (sucking and nail biting), have been recognized nationwide [15, 16]. Finally, the discrepancy may also be due to lead exposure in air, water and soil in different areas.

In 2008–2009, Etchevers A et al. demonstrated that the geometric mean of BLL was 14.9 μg/L and that the prevalence of BLLs exceeding 100 μg/L was 0.09% in a nationwide cross-sectional survey of children aged 6 months-6 years old in France [17]. Similarly, in the United States, the geometric mean of BLLs was 13 μg/L and the prevalence rate for confirmed EBLLs≥50 μg/L per 100 tested children was 2.6% in children aged 1–5 years according to the National Health and Nutrition Examination Survey 2007–2008 [18]. Obviously, the geometric mean BLL (64.24μg/L) in our study was higher than in Western countries. There are several possible explanations for this observation. First, the major source of lead exposure is household dust from deteriorating paint and tap water from lead branch pipes in western countries where the use of lead in paint and pipes was completely abolished in the 1970s [19]. However, the major lead sources in China may come primarily from the burning of fossil fuels; however, leaded petrol was not banned until 2000. Second, the prevalence of malnutrition, such as lower levels of calcium, iron, zinc and other elements, might increase the risk for enhanced absorption of ingested lead [19, 20]. With the improvement of living conditions, the intake of milk has improved the lack of trace elements in the diet, which may reduce intestinal lead absorption [20]. Finally, regional disparity, dietary habits, and ethnic difference may lead to the differences in BLLs and the prevalence of EBLLs [15, 21].

From the analysis, we found that boys had higher BLLs and a higher prevalence of lead poisoning than girls. The finding in our study is comparable to observations in the US and in Canada [22, 23]. The change may be due to several possible explanations. First, boys may be participated in more outdoors activities than girls, which results in higher lead exposure [24]. Second, boys are inclined to have behavioral patterns, such as being naughty; are active; and have worse health habits, which may lead to more lead absorption [25].

Both BLLs and the prevalence of EBLLs increased with aged from 0–6 years and reached a peak in children aged five or six years old. The phenomenon was consistent with previous studies in China [26, 27]. The explanation for this observation is that most of the primary Pb will be retained and more will continue to accumulate in the body tissues of children under conditions of continued exposure from inhalation of airborne Pb [28, 29] and a longer time line of ingestion and eat greater amounts [30, 31]. In addition, the heaviest lead-contaminated zone in air was 75–100 cm over ground, which is the same as the average height of pre-school and primary school children [32]. Finally, older children are more likely to participate in outdoor activities, which expose them to lead-contaminated environments. However, the BLLs and prevalence of EBLLs in children generally peaks approximately 18–30 months of age in the United States. This is likely because the major source of lead is household dust from deteriorating paint and some hand-to-mouth behaviors, such as crawling on the floor and putting hands and toys in the mouth, at that age [19]. Rahman A et al. demonstrated that the BLL in toddlers is associated with the BLL during pregnancy through the placenta and during breastfeeding [33]. Currently, experts in China strongly suggest that young couples who are ready to have children should have a blood lead measurement before pregnancy, which will lead to lower BLLs in pregnancy. This may be an important reason for the lower the blood lead level in the toddler group.

The findings show that from 2009 to 2013, 10.54% of the children in Hunan Province exceeded the Center for Disease Control and Prevention BLLs of concern (≥100μg/L). Moreover, the prevalence of EBLLs declined by 77% from 18.31% to 4.26% between 2009 and 2013. In China, compared with the studies in 1995–2003, both BLLs and the prevalence of EBLLs were lower in studies carried out after 2004 [12]. Li T et al. have shown that the prevalence of EBLLs(≥100μg/L) declined from 9.78% to 1.32% between 2004 and 2010 in China [34]. In Canada, the geometric mean BLL was 9 μg/L and fewer than 1% of children between 6 and 11 years old had EBLLs [35]. Kennedy BS et al. revealed that the prevalence of EBLL per 100 tested children decreased from 13.4 to 1.1 in Monroe County, 6.3 to 1.0 in New York State, and 7.6 to 0.6 in the United State between 1997 and 2011 [36].

The BLLs and prevalence of EBLLs have demonstrated a clear downward trend from 2009 to 2013, but the trend exhibited a slight fluctuation from 2010 to 2012 in our study. Regarding the downward trends in both BLLs and the prevalence of EBLLs, there are some explanations. First, the major cause of lower BLLs is attributable to government policies to decrease the amount of lead in air, dust and water. For example, leaded petrol has been completely banned in China since 2000, and heavy industry enterprises were closed down or rectified in China, which led to the reduction of lead emission. In addition, the Ministry of Environmental Protection formally promulgated “Clean Production Standards for the Lead Battery Industry” and implemented this policy in February 2009. Second, the risk factors of high blood lead levels, such as not washing hands before meals, eating paint, ink, or crayons, and frequently eating preserved eggs or canned food, have been recognized nationwide [37–39]. Furthermore, nutritional factors such as iron, zinc and calcium, are thought to play an important role in lead accumulation and poisoning [40, 41]. Therefore, parents pay more attention to children’s healthy eating and living habits, which has led to the reduction of lead exposure. Regarding the slight fluctuation from 2010 to 2012, this may be attributed to the lead-acid battery (LAB) industry. The production of lead-acid battery (LAB) goods, such as electric bikes, automobiles, and photovoltaic systems, dramatically increased in China. Moreover, China’s LAB industry is the world’s largest in terms of production and consumption, accounting for over 30% of global LAB output [42, 43] and using over 67% of China’s total Pb production [44]. However, the Ministry of Environmental Protection along with the National Development and Reform Commission jointly issued an environmental protection special action decree in March 2011. With the enforcement of the decree, 1930 LAB enterprises were completely shut down and 405 LAB enterprise operations were suspended for rectification. Therefore, the rapid development of China’ lead-acid battery industry may be responsible for a slight fluctuation of EBLLs from 2010 to 2012.

### Limitations

Our study has some limitations. First, all subjects were outpatients for regular checkups at the largest tertiary hospital. These children might be from families with higher income or more educated parents Thus, there is a selection bias in this study. Second, we were not able to collect confounding information, such as parents’ education and occupation, family income, drinking water sources, living location and so on, which previous studies have been shown to have a link to EBLLs.

### Conclusion

In conclusion, blood lead levels in children have declined substantially from 2009 to 2013 in Hunan Province in China; however, the prevalence of elevated lead levels in children remains unacceptably high compared to developed countries. Hence, the government should initiate measures to improve health behaviors, such as washing hands before eating and eating food enriched with iron, zinc and/or calcium.
